# Obstacle avoidance planning of space manipulator end-effector based on improved ant colony algorithm

**DOI:** 10.1186/s40064-016-2157-x

**Published:** 2016-04-23

**Authors:** Dongsheng Zhou, Lan Wang, Qiang Zhang

**Affiliations:** Key Laboratory of Advanced Design and Intelligent Computing, Ministry of Education, Dalian University, Dalian, 116622 China

**Keywords:** Improved ant colony algorithm, Space manipulator, Obstacle avoidance, Path planning

## Abstract

With the development of aerospace engineering, the space on-orbit servicing has been brought more attention to many scholars. Obstacle avoidance planning of space manipulator end-effector also attracts increasing attention. This problem is complex due to the existence of obstacles. Therefore, it is essential to avoid obstacles in order to improve planning of space manipulator end-effector. In this paper, we proposed an improved ant colony algorithm to solve this problem, which is effective and simple. Firstly, the models were established respectively, including the kinematic model of space manipulator and expression of valid path in space environment. Secondly, we described an improved ant colony algorithm in detail, which can avoid trapping into local optimum. The search strategy, transfer rules, and pheromone update methods were all adjusted. Finally, the improved ant colony algorithm was compared with the classic ant colony algorithm through the experiments. The simulation results verify the correctness and effectiveness of the proposed algorithm.

## Background

A space manipulator system is composed of system body (satellite) and its on-board manipulator. Since the manipulator system with gas thruster can fly or float free in the micro-gravity space environment, which expands the working space for the manipulator, so the manipulator system could instead astronauts engaged in a variety of extravehicular activities. Path planning of the manipulator system will become one of the main research directions in the area of space in the future (Yoshida and Wilcox 2008). In the space environment, space debris and cabin peripheral testing devices have the potential to be obstacles for space manipulator in the process of on-orbit operation, and the collisions occurred between the manipulator and the obstacles will not only interfere with on-orbit operation to complete the task, but also do harm to the manipulator system and operation personnel. Therefore, the obstacle avoidance path planning of space manipulator has very important research significance.

The main idea of the obstacle avoidance planning is designing an optimal path which can avoid all obstacles and meet certain targets from the starting point to the target point. For the path planning problem, a number of methods have been addressed, such as C-space method (Ping et al. 2009), A* search method (Kala et al. 2010), the improved artificial potential field method (Liu and Zhang 2014), neural networks (Duguleana et al. 2012) and so on, but they all have certain limitations. C-space method needs large computation. The calculation is more time consuming than the response of the manipulator which has limited its range of application in the area of the practical obstacle avoidance. Since the computational amount of A* search method will increase sharply with the increase of space dimension, it is difficult to satisfy its time and space requirements. The improved artificial potential field method is very suitable for dealing with dynamic obstacles, but it is easy to fall into local minimum point. During the process of searching path, the neural network method was easy to lose information. This caused it is difficult to find a feasible path to meet the constraints in a complex environment.

Ant colony algorithm has strong robustness and ability of distributed computing, and it is easy to be combined with other methods, but it also has some defects, such as slow convergence speed, easily falling into local optimum. In this paper, firstly we establish models for the space manipulator and the environment, and then transform the search strategy, transfer rules (Hao and Wang 2010) and pheromone update methods to improve the ant colony algorithm, and finally the improved ant colony algorithm is used to search the better obstacle avoidance path. Using this method, manipulator end-effector can avoid all the obstacles smoothly and its motion path is shorter than the path obtained by the basic ant colony algorithm.

## Models of manipulator and environment

**Fig. 1:**
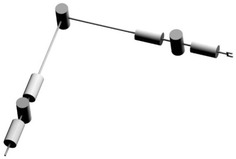
Structure diagram of S-R-S space manipulator

In this paper, we study the obstacle avoidance path planning of S-R-S space manipulator with seven degrees of freedom (Zhou et al. 2015). In the S-R-S structure, the first, second and third joint are used to compose the shoulder joint which can be equivalent to a virtual ball joint and its center is located in the intersection of three revolving axes, wrist joint is comprised of the 5th, 6th and 7th joint, the 4th joint is regarded as elbow joint which is a simple rotary joint. Figure 1 is the structure diagram of S-R-S space manipulator.

**Fig. 2:**
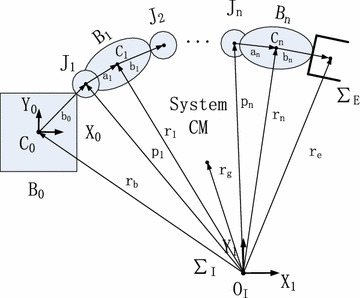
Structure diagram of general model

The description about joints information of S-R-S space manipulator can reference to the general model as Fig. 2. The D-H parameters of system and quality characteristics of space manipulator are shown as Tables 1 and 2. The comments of the above symbols are shown as Table 3.

**Table 1:** D-H parameters of system

*i*	$\alpha_{i - 1}$	$a_{i - 1}$	*d*_*i*_	*θ*_*i*_
1	$- \pi /2$	0	*d*_1_	*θ*_1_
2	$\pi /2$	0	0	*θ*_2_
3	$- \pi /2$	0	*d*_3_	*θ*_3_
4	$\pi /2$	0	0	*θ*_4_
5	$- \pi /2$	0	*d*_5_	*θ*_5_
6	$\pi /2$	0	0	*θ*_6_
7	0	0	*d*_7_	*θ*_7_

**Table 2:** Quality characteristics of space manipulator

	*B*_0_	*B*_1_	*B*_2_	*B*_3_	*B*_4_	*B*_5_	*B*_6_	*B*_7_
*m* (kg)	1146.342	3.692	60.416	3.678	35.654	3.461	2.624	0.114
*l* (m)	0.85	0.13	1.5	0.12	1.24	0.22	0.07	0.01
*I*_*xx*_ (kg m^2^)	291.077	0.013	13.556	0.012	1.235	0.061	0.066	0
*I*_*yy*_ (kg m^2^)	536.263	0.005	2.274	0.010	7.309	0.028	0.004	0
*I*_*zz*_ (kg m^2^)	669.647	0.015	15.756	0.005	6.104	0.035	0.004	0

**Table 3:** The comments of the symbols

System CM	The system’s center of gravity
$\sum {\text{I}}$	The inertial coordinate system
*B*_0_	Base of the system
*B*_*i*_ (*i* = 1,2…,7)	The *i*th link
$J_{i}$	The *i*th joint
$C_{i}$	Gravity center of *B*_*i*_
${\varvec{a}}_{i}$	Position vector from *J*_*i*_ to *C*_*i*_
${\varvec{b}}_{i}$	The distance from *C*_*i*_ to $J_{i + 1}$
${\varvec{b}}_{0} \in {\mathbf{R}}^{3}$	Position vector from CM of base to joint 1
${\varvec{r}}_{b} \in {\varvec{R}}^{3}$	Position vector of the center of mass (CM) of base
${\varvec{r}}_{i} \in {\varvec{R}}^{3} \left( {i = 1,2, \ldots ,7} \right)$	Position vector of CM of link $i$
${\varvec{r}}_{g} \in {\varvec{R}}^{3}$	Position vector of the system
${\varvec{r}}_{e} \in {\varvec{R}}^{3}$	Position vector of end-effector
${\varvec{p}}_{i} \in {\varvec{R}}^{3}$	Position vector of joint $i$
${\varvec{p}}_{i}$	Position vector of $J_{i}$
$\alpha_{i - 1}$	The link corner of manipulator
$a_{i - 1}$	The length of common vertical line from the joint shaft $i - 1$ to *i*
*d*_*i*_	The link offset
*θ*_*i*_	The *i*th joint angle
${\varvec{\varTheta}} \in {\varvec{R}}^{n}$	Joint angle vector
*m*_*i*_	Mass of link *i*
*l*_*i*_	Length of the *i*th link
*I*	Movement inertia of the links

### Kinematics equation of space manipulator

Japanese scholars Y. Umetani and K. Yoshida proposed the Generalized Jacobian Matrix which reflects the relationships between the motion velocity of end-effector and angular velocity of joints, Xu Wenfu etc. (Xu and Li 2008) analyzed the kinematics equation of free floating space manipulator based on Generalized Jacobian Matrix:

1 $$\left[ {\begin{array}{*{20}c} {\varvec{v}}_{e} \\ {{\varvec{\omega}}_{e} } \\ \end{array}}\right] = \left[ {\varvec{J}_{m} - {\varvec{J}}_{b} {\varvec{I}}_{b}^{ - 1} {\varvec{I}}_{M} } \right] \, {\dot{\varvec{\varTheta }}} = {\varvec{J}}^{*} \left({{\varvec{\Psi}}_{b} ,{\varvec{\varTheta}},m_{i} ,{\varvec{I}}_{i} } \right)\;{\dot{\varvec{\varTheta }}}$$

where, ${\varvec{v}}_{e}$, ${\varvec{\omega}}_{e}$ are respectively the linear and the angular velocity of manipulator end-effector, ${\varvec{J}}_{m}$ is a Jacobian matrix bound up with the motion of manipulator, ${\varvec{J}}_{b}$ denotes a Jacobian matrix associated with the base motion, ${\varvec{J}}_{bm}$ is a matrix associated with base and manipulator, ${\dot{\varvec{\varTheta }}}$ is a set of seven joint angles, ${\varvec{J}}^{*} \left( {{\varvec{\Psi}}_{b} ,{\varvec{\varTheta}},m_{i} ,{\varvec{I}}_{i} } \right)$ is the generalized Jacobian matrix of space manipulator, which is the function about base attitude, joint angles of manipulator, the mass, and inertia of rigid body. The kinematics equation is derived from the centroid position, the velocity of the links, the end-effector and the momentum conservation law, which is a mature and classic equation and already applied widely in many areas. The generalized Jacobian matrix is indispensable in path planning of space manipulator.

### Expression of valid path in space environment

**Fig. 3:**
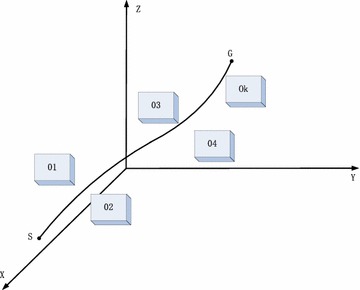
Bypass through the space obstacles

The task of obstacle avoidance path planning is to find a non-collision way from a starting position to the target location according to a certain evaluation standard in the environment with obstacles. We assume that *S* is the position of manipulator end-effector and *G* is the target position, and there are some obstacles between *S* and *G*. Manipulator will search a short and safety path from *S* to *G*, and it is shown in Fig. 3.

**Fig. 4:**
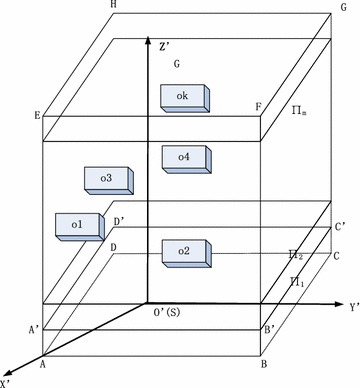
Illustration of space obstacles in coordinate $O^{\prime} - X^{\prime}Y^{\prime}Z^{\prime}$

For the convenience of the research, we established the coordinate $O^{\prime} - X^{\prime}\,Y^{\prime}\,Z^{\prime}$ shown in Fig. 4, where, $S$ is the origin point in the new coordinate, the $SG$ direction is forward $Z^{\prime}$ axis, and the direction of $X^{\prime}$ and $Y^{\prime}$ could be chosen properly. The transformation between the coordinate system of $O^{\prime} - X^{\prime}Y^{\prime}Z^{\prime}$ and $O - X{\kern 1pt} YZ$ can be described as the following formula (Porta Garcia et al. 2009):

2 $$\left[ {\begin{array}{*{20}c} x \\ y \\ z \\ \end{array} } \right] = \left[ {\begin{array}{*{20}c} {\cos \alpha_{x} } & {\cos \alpha_{y} } & {\cos \alpha_{z} } \\ {\cos \beta_{x} } & {\cos \beta_{y} } & {\cos \beta_{z} } \\ {\cos \gamma_{x} } & {\cos \gamma_{y} } & {\cos \gamma_{z} } \\ \end{array} } \right]\left[ {\begin{array}{*{20}c} {x^{\prime} } \\ {y^{\prime} } \\ {z^{\prime} } \\ \end{array} } \right]$$

where, $\alpha_{x}$, $\beta_{x}$ and $\gamma_{x}$ are the intersection angle between $X$ axis and $X^{\prime}$, $Y^{\prime}$, $Z^{\prime}$. $\alpha_{y}$, $\beta_{y}$ and $\gamma_{y}$ are the intersection angle between *Y* axis and $X^{\prime}$, $Y^{\prime}$, $Z^{\prime}$. $\alpha_{\text{z}}$, $\beta_{z}$ and $\gamma_{z}$ are the intersection angle between $Z$ axis and $X^{\prime}$, $Y^{\prime}$, $Z^{\prime}$. The coordinates of the obstacles can be calculated by the formula (2).

**Fig. 5:**
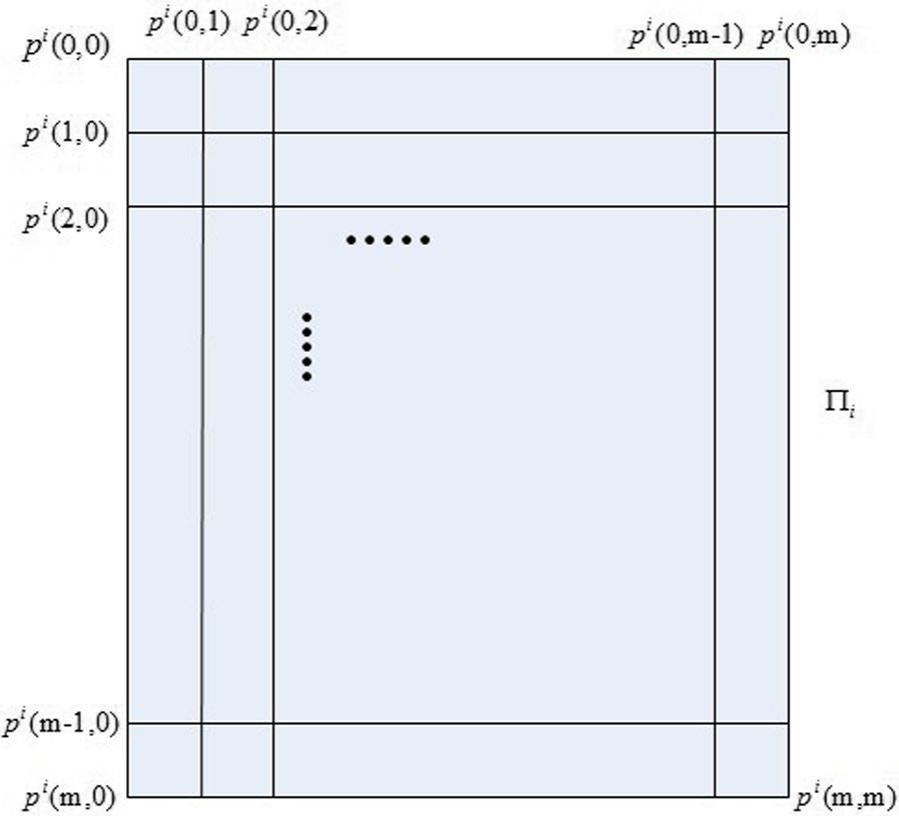
Representation of grid coordinate in plane $\prod_{i}$

As shown in Fig. 4, we established a cube $ABCDEFGH$ in the coordinate $O^{\prime} - X^{\prime}Y^{\prime}Z^{\prime}$, and the face $ABCD$ of the cube is in plane $X^{\prime}{\kern 1pt} Y^{\prime}$. In this paper, the method of equally dividing space was used to extract grid points that the path planning needed from the three-dimensional space. Firstly, $ABCDEFGH$ is divided equally along the border $AB$, getting $n + 1$ planes, then the planes are divided into $m$ and $l$ parts equally along the border $AD$ and $AA^{\prime}$ in proper sequence, and the intersection point inside can be resolved (Hu and Cai 2011). Plane partition is shown in Fig. 5.

## Obstacle avoidance planning based on improved ant colony algorithm

Inspired by the fact that ants always find a shortest path between the food and the nest during the foraging process, M. Dorigo proposed ant colony algorithm (Dorigo et al. 1996). Ant colony algorithm has many advantages, but there are some defects, such as high complexity, easy to fall into local optimum. In order to obtain the better obstacle avoidance path, the search strategy, transition rule and pheromone updating method in classical ant colony algorithm are improved in this paper.

### Visual area

In this paper, we take *x* axis for the main direction of path planning and set the maximum transverse and longitudinal moving distance for ants (Feng et al. 2011). Thus, there is a visible area when the ants search the next node. This area plays the role of simplifying the search space and improving the search efficiency of ant colony algorithm.

### Fitness function

With the purpose of searching the shortest obstacle avoidance path between starting point and the target point, the fitness function is defined as:

3 $$F\left( {x,y,z} \right) = \sum\limits_{i = 1}^{n} {\sqrt {1 + \left( {y_{i + 1} - y_{i} } \right)^{2} + \left( {z_{i + 1} - z_{i} } \right)^{2} } }$$

where, $n$ is the number of populations, $i$ means the current point, and $i + 1$ means the next point.

### Search strategy

The selection probability of each point is calculated according to the heuristic function when ants move from the current point to next point, the heuristic function in this paper is defined as (Sgorbissa and Zaccaria 2012):

4 $$H\,\left( {i,j,k} \right) = D\,\left( {i,j,k} \right) \cdot S\,\left( {i,j,k} \right) \cdot Q\,\left( {i,j,k} \right)$$

where, $D\,\left( {i,j,k} \right)$ represents the distance between two points, making the ants move towards its near point, its expression is shown as follows:

5 $$D\,\left( {i,j,k} \right) = \sqrt {\left( {x_{a} - x_{b} } \right)^{2} + \left( {y_{a} - y_{b} } \right)^{2} + \left( {z_{a} - z_{b} } \right)^{2} }$$

where, $a$, $b$ are the expression of the current and next point respectively.

$S\,\left( {i,j,k} \right)$ denotes the security factors impelling ants to select safe points, and we use $Num$ and $UNum$ to show:

6 $$S\left( {i,j,k} \right) = \frac{Num - UNum}{Num}$$

where, $Num$ denotes the number of viewpoints, $UNum$ indicates the number of unreachable points in viewpoints region. When the chosen point cannot be reached,

7 $$S\,\left( {i,j,k} \right) = 0$$

$Q(i,j,k)$ is the length of path from next point to target point, which makes ants choose the points closer to the goal point, and its expression is given as follows:

8 $$Q(i, j,k)= \sqrt{(x_{b}-x_{d})^{2}+(y_{b}-y_{d})^{2}+(z_{b}-z_{d})^{2}}$$

where, *b*, *d* denotes the next and target point respectively.

The steps that ants select next point $p_{i + 1}$ in plane $\prod_{i + 1}$ are as follows:

- Step 1: Determine the set of feasible points in the plane $\prod_{i + 1}$.
- Step 2: Calculate the heuristic information value $H_{a + 1,u,v}$ of the set of feasible points according to the formula (4).
- Step 3: Calculate the selection probability $p\,\left( {i + 1,u,v} \right)$ at any point $\left( {i + 1,u,v} \right)$ within the plane $\prod_{i + 1}$:
- 9 $$p\left( {i + 1,u,v} \right) = \left\{ {\begin{array}{ll} \frac{\tau_{a + 1,u,v} H_{a + 1,u,v}} {\sum (\tau_{a + 1,u,v} H_{a + 1,u,v})} & {at\,feasible\,points} \\ 0 & {others} \\ \end{array} } \right.$$
- where, $\tau_{a + 1,u,v}$ is the pheromone of point $p\,\left( {a + 1,u,v} \right)$ in the plane $\prod_{i + 1}$.
- Step 4: Select the points in plane $\prod_{i + 1}$ using the roulette wheel method on the basis of the selection probability of each point.

### Transition probability

The probability value is computed by formula (9) during the process that ants search the obstacle avoidance path, and the node with the highest probability is selected as the next node. Due to the fact that ant colony algorithm is easy to fall into local optimum, the chosen node with high probability may not be the optimal solution, if the size of problem is large, it is more difficult to find the optimal solution (Hao and Wang 2010). In addition, if transfer probability is only obtained by using the (9), the algorithm will lose randomness and some good solutions will be ignored. Then, the consequence is that the global optimal solution is wrong. Therefore, the method of combining random and deterministic probability is adopted to select the nodes.

To adjust the degree of exploring a new path and make the search activities focus on the spatial neighborhood of the optimal solution, the parameter $q_{0}$ is introduced in this paper. If an ant is located at the node $i$, it will choose the next node to transfer according to the following formula:

10 $$k = \left\{ {\begin{array}{*{20}l} {\arg \,\hbox{max} \left\{ {\tau_{a + 1,u,v} H_{a + 1,u,v} } \right\}} \hfill & {if\,\,q \le q_{0} } \hfill \\ j \hfill & {else} \hfill \\ \end{array} } \right.$$

where, $\tau_{a + 1,u,v}$ is the pheromone value of the point $p\,\left( {a + 1,u,v} \right)$ in plane $\prod_{i + 1}$, and $H_{a + 1,u,v}$ denotes the heuristic information value of the set of feasible points from the point $p_{i}$ to the points in plane $\prod_{i + 1}$. $q$ is the random number in the evenly distributed interval $\left( { 0 , 1} \right]$, and $q_{0}$ is the number in the interval $\left( { 0 , 1} \right]$, we define the value of $q_{0}$ is 0.5 in the path planning. $j$ is the selected node according to the formula (10).

### Updating pheromone

In order to increase the probability of the points that ants have not passed in the search process and achieve global search, local pheromone updating as ants search, and the updating formula is:

11 $$\tau_{ijk} = \left( {1 - \varsigma } \right)\,\tau_{ijk}$$

In the above formula, $\tau_{ijk}$ is the pheromone value at $\left( {i,j,k} \right)$, $\varsigma$ indicates the attenuation coefficient of pheromone.

Global update is to regard the length of path which ants have finished searching as the evaluation value and select the shortest path from the set of path. Increasing the pheromone of nodes in the shortest path, updating formulas are shown as follows (Dong et al. 2009):

12 $$\tau_{ijk} = \left( {1 - \rho } \right)\,\tau_{ijk} + \rho \,\Delta \tau_{ijk}$$

13 $$\Delta \tau_{ijk} = \frac{K}{{\hbox{min} \,\left( {length\,\left( m \right)} \right)}}$$

where, $\rho$ is the pheromone update coefficient, $K$ is a constant, $length\,\left( m \right)$ shows the length of path that the *m*th ant has passed.

### Steps of the algorithm

**Fig. 6:**
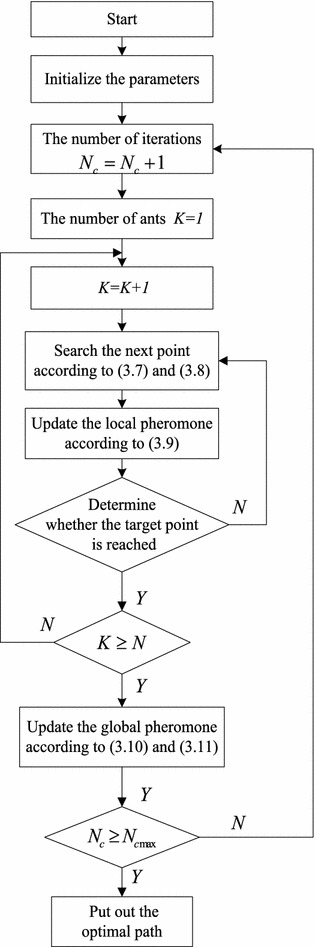
Flow chartoftheimproved ant colony algorithm

The progress of improved ant colony algorithm is shown in Fig. 6.

- Step1: Initialization. The ants whose number is $m$ are placed in the start point, setting iteration counter $N_{c} = 0$, the maximum number of iterations $N_{c{\rm max}}$, the number of ants $N$, attenuation coefficient and update coefficient of the pheromone.
- Step2: Ants search path. Search the next point according to formula (9) and (10), at the same time, record the path that ants have walked and update the local pheromone according to the formula (11).
- Step3: To determine whether the target point is reached, then go to Step4, otherwise turn to Step2.
- Step4: Update the total length of path. If the new path is shorter than the length of the known optimal path, we will replace the original optimal path with a new path.
- Step5: Update the global pheromone for the updated path base on (12) and (13).
- Step6: $N_{c} = N_{c} + 1$, if $N_{c} \le N_{c{\rm max} }$, then turn to Step2, otherwise put out the optimal path $p$.

## Simulation results

In this paper, the space manipulator with seven degrees of freedom is used for path planning about obstacle avoidance, for the convenience of theoretical research, the end-effector is only considered instead of the whole manipulator system.

In our experiments, three obstacles are set up and each obstacle is irregular body whose surface and bottom are both parallel to the plane *XY*. The eight vertex coordinates of the first obstacle are: (4,4,1), (4,8,1), (8,4,1), (8,8,1), (5,5,4), (7,5,4), (5,7,4), (7,7,4); The second obstacle’s coordinates are: (10,8,1.25), (10,12,1.25), (14,8,1.25), (14,12,1.25), (11,9,5), (11,11,5), (13,9,5), (13,11,5); The eight vertex coordinates of the third obstacle are: (16,12,1.5), (16,16,1.5), (20,12,1.5), (20,16,1.5), (17,13,6), (17,15,6), (19,13,6), (19,15,6). The coordinates of starting point and target point are (1, 4, 2) and (21, 14, 1), respectively. Parameters of the improved ant colony algorithm are shown in Table 4.

**Table 4:** Parameters setting of improved ant colony algorithm

Size of population	Decay coefficient of pheromone	Update coefficient of pheromone	Number of iterations	Coefficient K
20	0.9	0.2	100	100

In the process of simulation for the improved ant colony algorithm, it is easy to find out that the path ants have walked is different every time under the same program. Therefore, ant colony algorithm and the improved ant colony algorithm were both run 100 times, and then we compared their average fitness value, operation time and optimal fitness values separately. The results are shown in Table 5.

**Table 5:** Comparisons between ant colony algorithm and improved ant colony algorithm

	Average fitness (m)	Average time (s)	Optimal fitness (m)
Ant colony algorithm	62.1348	9.9841	58.9486
Improved ant colony algorithm	55.2767	9.6831	50.7498

**Fig. 7:**
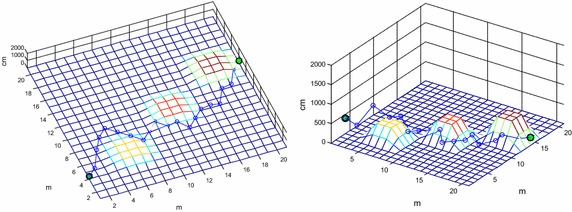
Obstacle avoidance path obtained by ant colony algorithm in different viewpoints

**Fig. 8:**
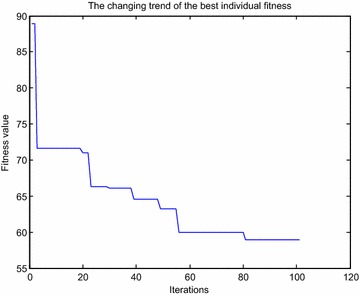
Variations of fitness obtained by ant colony algorithm

An obstacle avoidance path shown in Fig. 7 is obtained by ant colony algorithm, whose optimal fitness value is 58.9486 m, and its variations of fitness is shown in Fig. 8.

**Fig. 9:**
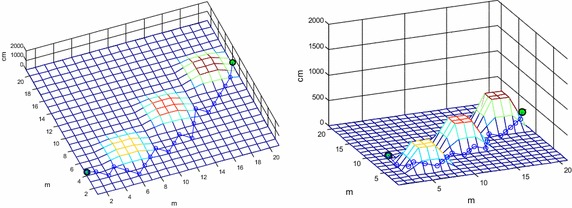
Obstacle avoidance path obtained by improved ant colony algorithm in different viewpoints

**Fig. 10:**
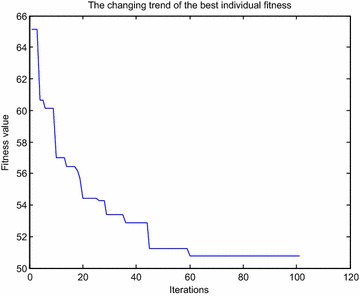
Variations of fitness obtained by improved ant colony algorithm

The optimal obstacle avoidance path and the variations of fitness shown in Figs. 9 and 10 are both obtained by improved ant colony algorithm.

The idea that choosing the next node with probability is similar to the greedy algorithm, they all consider the local optimal, but the ant colony algorithm considers the distance from the next node to the target, which can avoid falling in local optimum.

The fitness obtained by improved ant colony algorithm is smaller than ant colony algorithm, that is to say, the obstacle avoidance path achieved by improved ant colony algorithm is shorter than ant colony algorithm; Ant colony algorithm reaches the optimal fitness value close to 80 iterations, while improved ant colony algorithm only need more than 60 iterations, which shows that the convergence rate of improved ant colony algorithm is faster than ant colony algorithm; From the point of the whole running time, the average time of improved ant colony algorithm is shorter than ant colony algorithm. The above results show that the avoidance effect obtained by the presented algorithm is more effective than the standard ant colony algorithm for manipulator end-effector.

## Conclusions

In this paper, an improved ant colony algorithm was presented and used for to plan the obstacle avoidance path to avoid the collisions between the space manipulator and obstacles when the manipulator executes the on-orbit service. The transfer rules, pheromone update method and search strategy of the ant colony algorithm were discussed and improved. Compared with the standard ant colony algorithm, experiments show that the presented algorithm has many advantages: the efficiency of path planning is increased, and the planned path is shorter and safer. Simulation results demonstrate the feasibility and effectiveness of this method.

Of course, there still exist many aspects that need to be studied deeply. And, it will be a challenge to deal with a more complexity obstacles layout or a dynamic unstructured environment which will be our next most important research point.
